# Projecting the Impact of Regional Land-Use Change and Water Management Policies on Lake Water Quality: An Application to Periurban Lakes and Reservoirs

**DOI:** 10.1371/journal.pone.0072227

**Published:** 2013-08-26

**Authors:** Arnaud Catherine, David Mouillot, Selma Maloufi, Marc Troussellier, Cécile Bernard

**Affiliations:** 1 Laboratoire Molécules de communication et adaptation des micro-organismes, Muséum national d’Histoire naturelle, Paris, Ile-de-France, France; 2 Laboratoire Écologie des systèmes marins côtiers, Université de Montpellier 2, Montpellier, Hérault, France; University of Florida, United States of America

## Abstract

As the human population grows, the demand for living space and supplies of resources also increases, which may induce rapid change in land-use/land-cover (LULC) and associated pressures exerted on aquatic habitats. We propose a new approach to forecast the impact of regional land cover change and water management policies (i.e., targets in nutrient loads reduction) on lake and reservoir water eutrophication status using a model that requires minimal parameterisation compared with alternative methods. This approach was applied to a set of 48 periurban lakes located in the Ile de France region (IDF, France) to simulate catchment-scale management scenarios. Model outputs were subsequently compared to governmental agencies’ 2030 forecasts. Our model indicated that the efforts made to reduce pressure in the catchment of seepage lakes might be expected to be proportional to the gain that might be obtained, whereas drainage lakes will display little improvement until a critical level of pressure reduction is reached. The model also indicated that remediation measures, as currently planned by governmental agencies, might only have a marginal impact on improving the eutrophication status of lakes and reservoirs within the IDF region. Despite the commitment to appropriately managing the water resources in many countries, prospective tools to evaluate the potential impacts of global change on freshwater ecosystems integrity at medium to large spatial scales are lacking. This study proposes a new approach to investigate the impact of region-scale human-driven changes on lake and reservoir ecological status and could be implemented elsewhere with limited parameterisation. Issues are discussed that relate to model uncertainty and to its relevance as a tool applied to decision-making.

## Introduction

Lakes and reservoirs provide critical goods and services, among which water provision, food production, and flood regulation are essential to supporting human needs [1]. The functioning of these freshwater ecosystems is tightly linked to external conditions operating from local to global scales [2] and is subject to multiple stressors arising from human activity and long-term change in background conditions [2]–[5]. Among the external stressors affected by global changes, climate change remains the most studied phenomenon that will most likely impact freshwater ecosystems in future decades (Figure S1). However, without disregarding the impact of increased temperature, changes in nutrient concentrations reaching aquatic ecosystems should be considered as a consistently more important driver of poor water quality [6], [7]. As the human population grows, so does the demand for living space and basic resources (food and water) required to meet human needs [8], which in turn leads to aquatic ecosystem degradation [9]. In a report on world urbanisation prospects, the UN [10] indicated that the worldwide urban population should steadily increase in the next decades (+40% by 2030), which will further increase anthropogenic pressures on aquatic ecosystems. Change in land-cover and intensification of land-use will thus likely impact natural habitats far more rapidly than the slow-acting climate change and will deeply modify freshwater species assemblages and ecosystem functioning [11].

Following binding legal instruments, such as the Water Framework Directive (WFD) [12], requiring European freshwaters to be brought to good ecological status (GES), increasing efforts and resources are being dedicated to improving freshwater habitat quality. However, despite the re-oligotrophication efforts in several regions [13], cultural eutrophication remains one of the primary reasons for failing to achieve a GES by 2015 and beyond [14], [15]. It also results in a significant reduction of freshwater ecosystems economic and amenity value (e.g., increased cost of water treatment and impact on the tourism industry) [16], [17]. Over 50% of European lakes and reservoirs may experience significant eutrophication, and this state may exceed 40% in most areas of the world [18]. Despite a significant decrease in the 1990s, phosphorus and nitrogen concentrations in the rivers and lakes of Western Europe appear to be relatively stable since then [19]. However, unless further nutrient reduction policies are established, the consequences of the projected increase in human population may counteract the current eutrophication mitigation policies. In this context, management tools aiming to forecast the efficiency of water management policies (i.e., targets in nutrient loads reduction) on lake and reservoir water quality and taking into account projected land-use/land-cover (LULC) and population changes are highly desirable.

To date, the published scenarios focused on freshwater ecosystems have primarily been designed to evaluate the impact of nutrient reduction policies [13] and/or climate change [6], [20] on the water quality and ecological state of water bodies and on species range shifts and biodiversity patterns [21], [22]. Most scenarios have focused on predicting trends of lake eutrophication status (chlorophyll *a*) in response to a change in nutrient inputs and rely on linear models using TP as the main predictor [23]–[25], ecosystem scale mass-balanced models [26], [27], and process-based models [28]. To relate land-use to nutrient loading, these models require an estimation of nutrient export coefficients (NECs) for various land-use classes. However, the main limitation of NECs is their dependency on local factors (e.g., precipitation, agricultural practices, waste-water management) [29]–[30], which limits their transferability. NECs are ideally obtained from intensive water quality studies in catchments dominated by a single land-use class [29], and estimating regionally specific NECs often appears to be too resource-intensive to be applied to large-scale studies.

To overcome this issue, a number of studies have assessed the ability of catchment-scale variables to provide valuable information to understand and predict the spatial distribution of eutrophicated water bodies in the landscape [31], [32]. Though atmospheric deposition may have a significant impact on unproductive lakes located in undisturbed areas [33], the eutrophication status of most water bodies is a direct consequence of the quantity and quality of input waters, which is, in turn, directly dependent on their catchment characteristics. For instance, *all else being equal*, catchment size will likely be correlated to water fluxes and thus to nutrient fluxes reaching a given water body [34], [35]. However, in heterogeneous landscapes, a number of variables will also be influential and may modulate the transfer of nutrients into water bodies. Among these variables, drainage intensity (i.e., waterway length per unit of surface area) will influence water transport within catchments and thus the nutrient loadings reaching water bodies [36]. Likewise, the location of lakes and reservoirs in the landscape will determine those that are essentially receiving run-off waters (high altitude) and those that are influenced by free surface aquifer and flooding events (low-altitude flood plain) [37]. Finally, land-use is also a major factor affecting both the quantity and quality of nutrient loadings [31], [36]. Indeed, a number of studies have put forward a relation between land use and NECs [31], [38]–[40]. These differences in NECs mainly originate from differences in soil permeability among LULC classes and from land management practices within LULC classes. Agricultural activities are known to be major contributors of non-point source (NPS) pollutants through various processes (nutrients from the use of fertilisers and/or animal wastes, suspended sediments from frequent tillage, high surface runoff yield originating from tile drainage, and other such sources) [41]–[43]. However, nutrient loads originating from urban catchments may exceed that from agriculture-dominated catchments [39], [44] as the low permeability of urban LULC increase their runoff compared with other LULC classes.

The relationships between the catchment scale characteristics and the eutrophication status of water bodies are likely to be non-linear because land-use effects on nutrient contents exhibit threshold responses [32], [40]. In addition to non-linear relationships, complex interactions among predictors might be expected in such systems where different catchment characteristics may interact to drive nutrient fluxes reaching water bodies and thus influence their eutrophication status [32].

In this paper, we propose a general approach to forecasting the impact of regional LULC change and water management policies (i.e., targets in nutrient loads reduction) on lake and reservoir water quality using a model that requires minimal parameterisation compared with alternative methods [32]. This approach was applied to a set of 48 periurban lakes and reservoirs located in the Ile-de-France region (IDF, France) to simulate catchment-scale management exploratory scenarios. Model outputs were subsequently compared with a 2030 policy scenario defined from governmental agencies’ forecasts.

## Materials and Methods

### Random Forest (RF) Model

Chlorophyll *a* (Chl*a*) was used as an indicator of lake water eutrophication status as it is directly related to nutrient loadings that reach water systems [24], [45]. Chl*a* was predicted as a function of catchment-scale landscape and hydromorphic variables and water body characteristics using Random Forest (RF, R package ‘randomForest’) [46], [47]. RF is an ensemble learning method that builds a collection of classification or regression trees. Trees are built using a bootstrap sample of the observations and a random set of potential predictors (i.e., explanatory variables) to determine the best split at each node. Trees are grown to maximum size without pruning, and aggregation of trees is performed by averaging.

In a previous study, we demonstrated that RF provides a better prediction accuracy over more conventional statistical modelling techniques such as generalised linear models (GLM) or generalised additive models (GAM) [32].

Two RF models were built: one allowing the prediction of Chl*a* as a continuous response variable and the other considering Chl*a* as a binomial variable. In the binomial model, the threshold Chl*a* concentration was set to 25 µg L^−1^, which corresponds to a state of significant eutrophication in the Organisation for Economic Cooperation and Development (OECD) fixed boundary system [24].

### Model Parameterisation

A set of catchment and water body characteristics (Table S1) was used to parameterise the RF model on the basis of their known contribution to nutrient loadings and water body buffering capacities (see [32] for details). The ratio between catchment and water body size (S) was used as a surrogate of nutrient loading [32], [34], [35], [48]. The percentages of land covered by the main LULC classes (forest: LF, agricultural: LA, and impervious: LI) were also included as explanatory variables because they constitute well-known variables acting on both the quantity and quality of nutrient loadings [31], [36]. The catchment capacity to transport nutrients was taken into account using the density of the drainage network (Id) [36], [49].

Additional water body characteristics were also used as predictors. Lake mean depth (D) was included in the model as it modulates the buffering capacity of nutrient loadings for a given catchment to water body size ratio [31], [49] and also affects primary production through processes such as light penetration into the water column, water column mixing, and sedimentation processes [50]. In addition, the altitude of water bodies was included in the RF model as it informs (i) whether a given water body is located in a stream floodplain, which may significantly increase nutrient loadings during unusual hydrological events and (ii) whether the water body is influenced by free surface aquifers, which may significantly act on the water level of seepage lakes during low rainfall periods [37]. Finally, season (T), a parameter that influences the timing and extent of phytoplankton growth [51], was included in the model. All explanatory variables were normalised and included in the RF model without weighting. All GIS analyses were performed using MapInfo 8.0 Professional® (MapInfo Inc., Troy, Michigan, USA).

### Study Area and Sampling

**Figure 1:**
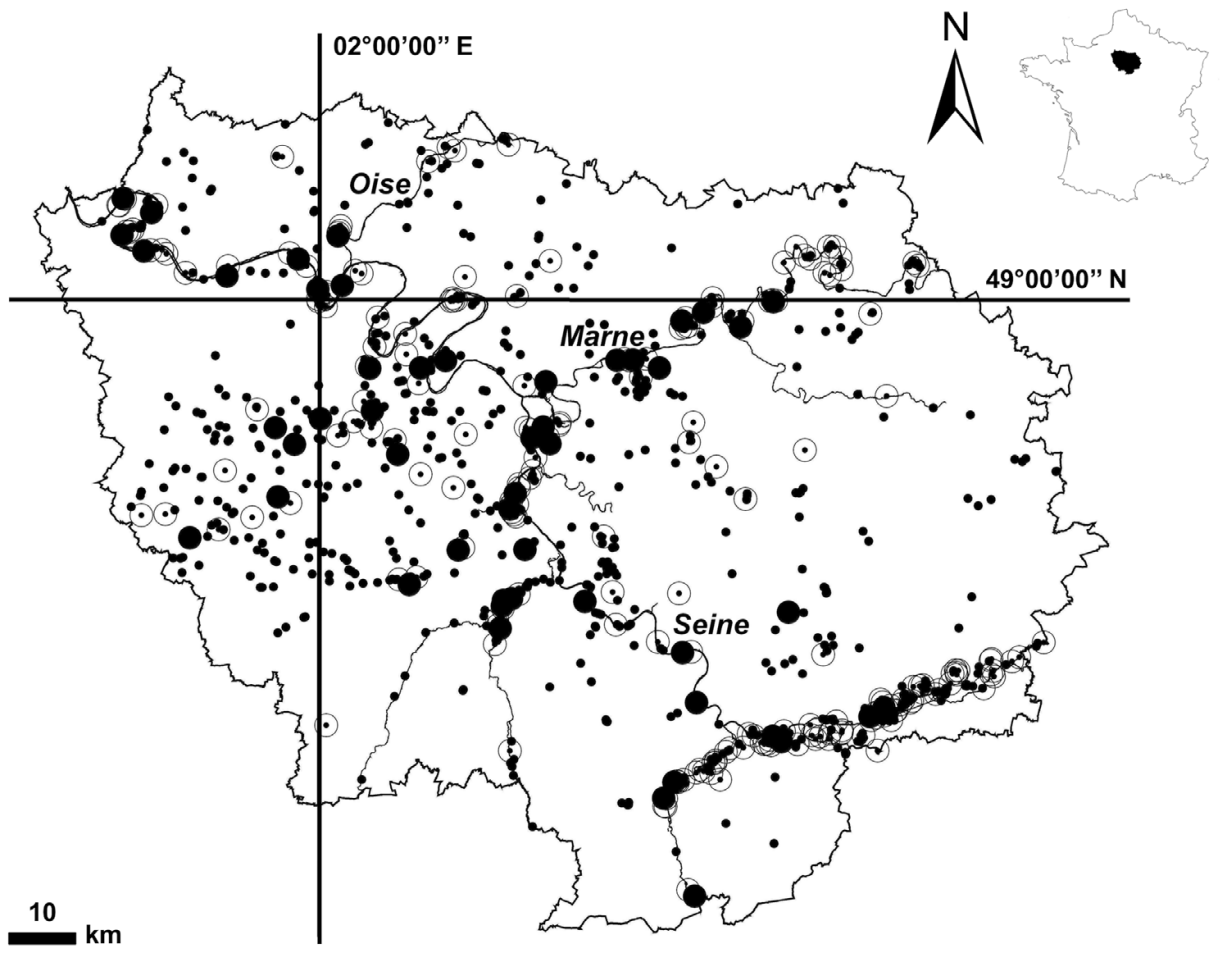
Map of the Ile-de-France region (inset showing location within France) showing the location of the 980 lakes, reservoirs and ponds included in the Carthage 3.0® GIS database (small black circles; n = 980), the water bodies covering >5 ha (open circles; n = 248) and the sampled water bodies (large black circles; n = 48).

The modelling approach presented above was applied to the Ile-de-France region (IDF; Figure 1). The IDF region covers a 12,011 km^2^ area surrounding Paris (north-central France). It is a fertile sedimentary plateau lying within a single first-order hydro-ecoregion [52]. Differences in terms of climate and geology, which are not accounted for in the model, are considered to be minor [53]. The IDF region displays strong LULC gradients and includes a wide range of lakes and reservoirs [54]. Thus, it constitutes a good mesoscale study area to develop statistical modelling of the impact of anthropogenic pressure change on lake and reservoir water quality.

Among the 248 water bodies covering more than 5 ha and recorded in the hydrological database Carthage 3.0® (IGN, Paris, France), 48 lakes and reservoirs (Figure 1) were chosen using a stratified sampling strategy previously described [54]. They were selected to represent an unbiased set of water bodies that reflects the whole range of environmental conditions (land use, hydrology, altitude, etc.) found within the IDF region. All of the water bodies sampled in this study can be considered as shallow according to the definition of Scheffer [55]. Four sampling field campaigns (one by season) were conducted (see [32] for details). All necessary permits were obtained to access the sampled lakes.

Chl*a* was measured using a FluoroProbe II (FP) *in situ* fluorometer (bbe-Moldaenke, Kiel, Germany). This device uses the spectral fluorescence approach to quantify phytoplankton biomass (expressed in µg.L^−1^ of Chl*a*). It allows a selective excitation of accessory pigments and measures the subsequent emission of fluorescence by reaction centre chlorophyll *a*. FP data were in good agreement with Chl*a* data obtained from a spectrophotometric method and well correlated to phytoplankton biovolume data [56]. For each lake, vertical profiles were obtained at 3 sampling stations to account for both vertical and horizontal heterogeneity.

### Model Validation

The predictive power of the RF model was assessed through a cross-validation procedure using the IDF data. To avoid circularity, two independent data sets were defined for model calibration and validation [57]. This technique was applied by fitting the models using a random subset (80%) of the data and testing the prediction accuracy using the remaining data (20%). This procedure was repeated 30 times, and the predictions obtained were averaged. The accuracy of models for predicting Chl*a* values was assessed by averaging R^2^ values between the observed and predicted values, whereas the goodness of prediction of Chl*a* binomially encoded was assessed using averaged Cohen’s Kappa values [58]. Following a cross-validation, the models were shown to yield high prediction accuracy for both continuous (phytoplankton biomass, R^2^ = 0.715) and binomial (eutrophic or not; kappa = 0.764) response variables [32].

### Definition of a 2030 Policy Scenario for the IDF Region

Local governmental agencies involved in water resource management plan to achieve a significant reduction of nutrient inputs reaching hydrosystems in the near future. The Seine-Normandie Water Agency, based on the outputs of the SENEQUE model [59], forecasts a 30–50% overall reduction of macropollutants (organic matter, nitrogen, and phosphorus) discharge compared to the levels of the year 2000 [60]. This reduction is expected to originate from a better treatment of point source pollution by (i) increasing water treatment investments, (ii) reducing the impacts of industrial activities through political and societal pressure and (iii) converting cattle farms into cereal and sugar beet farming areas (primarily for biofuel production) and increasing the treatment efficiency of effluents originating from the remaining cattle farms [60]. Diffuse pollution should also be reduced by 30%, primarily through the adoption of good farming practices (e.g., reduction of root level concentration of fertilisers) [60]. Thus, according to current knowledge and considering a conservative figure, the overall nutrient fluxes reaching water bodies should be reduced by approximately 30% but with a qualitative change towards urban discharges by 2030.

However, in the meantime, the urban population in the IDF region is expected to have increased by 20% by 2030 [10], which was not considered by the SENEQUE model. This demographic increase will be accompanied by the building of 1.5 million new residences [61]. Néméry et al. [62] demonstrated that population density and impervious surface (LI) within the IDF region are highly correlated (R^2^ = 0.99, p<0.001 in the outer IDF region and R^2^ = 0.71, p<0.001 if the city of Paris is included). Thus, if no major changes occur in terms of urban planning, the impervious area is expected to increase by approximately 20% by 2030.

### Modelling Anthropogenic Pressure Changes

The projected demographic change was proxied by the percentage of urban cover (LI). Because no information regarding demographic change at the catchment scale was available, we modelled variations of LI coverage at the regional scale. Variation in the cover of impervious surface within catchments obviously leads to variation in agricultural cover (LA) and natural cover (LF). As the region is characterised by high housing prices and by forested area mainly used for agroforestry (and thus with low patrimonial value), we chose to keep the ratio between LA and LF constant.

The expected changes in nutrient fluxes resulting from the changes in water treatment efficiency were proxied using the ratio between catchment and water body size (S).

The scenario for the IDF region described above is based on the best available knowledge but may be subject to significant uncertainty. Thus, for both variables (LI and S), we simulated exploratory scenarios from a zero-impact hypothetical situation to twice their current state, using 2% increment steps. This procedure allowed analysing the (i) deviance from the scenario and (ii) the shape of the relationship among response variables to changes in both LI and S.

Preliminary analyses indicated that drainage and seepage water bodies responded differently to changes in external pressures, and model outputs were considered separately for each subset of lakes. All of the analyses described above were performed in the statistical environment R version 2.14.0 [63].

## Results and Discussion

### On the Use of Statistical Models to Develop Lakes Water Quality Regional Management Tools

Studies focusing on a single hydrological system offer the possibility to integrate complex models linking catchment management practices, hydrology and the ecological response of the target ecosystem. However, the main factor that limits their application to large-scale studies is the necessity of gathering detailed information to calibrate model parameters [64]. Alternative approaches requiring less detailed prior knowledge and using hybrid or fully statistical models have been successfully applied to assess ecosystems responses to environmental changes [65]–[66]. However, most of these studies have focused on single or few catchments.

Whereas water catchments are recognised as the optimal geographical area linking environmental processes to human impacts on the landscape [67], decisions regarding LULC and water quality management often target larger spatial scales. In addition, if science is going to be involved in providing management advice, the scale of study needs to match the scale at which management options are defined [64], [68]–[69]. Considering the limited resources available, modelling tools using GIS-based indicators and applied to project the impact of management options at large spatial scales may represent valuable tools for stakeholders. Though the objective of our approach is not to predict with great precision the *dynamics* of lake water quality of a given lake, this space-for-time approach using catchment-scale descriptors has been shown to allow prediction with a good accuracy of the eutrophication status of a large array of lakes at a regional scale [32]. The model was developed using an unbiased set of lakes within the study area and thus provides a relevant image of the state of lakes within the region. Despite the known limitations of our statistical model (lack of mechanistic description of processes, use of proxy variables), its advantage lies in minimal input data requirements.

### Catchment-scale Indicators of Anthropogenic Pressure Changes

**Figure 2:**
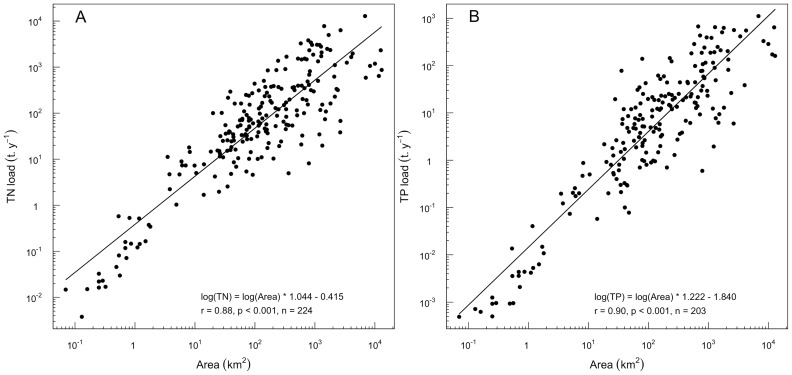
Relationship between total nitrogen (TN) and total phosphorus (TP) loads and catchment size in lakes and reservoirs (data from [61]–[67]).

The model used in this study includes environmental indicators that can be directly linked to change in environmental pressures. Based on expert knowledge [60], the increase in human population and the changes in nutrients loads following improved management of diffuse and point sources are considered to be the main factors that are likely to influence water quality within the IDF region in the coming decades. Changes in population density region were modelled using the percentage of impervious land cover within catchments, as available data indicated that both variables are linearly correlated in the study region [62]. Then, we modelled changes in nutrient loads using the ratio between catchment area and lake size. Using published data [70]–[74] originating from various geographic areas (Finland, The Netherlands, UK, and the USA), we demonstrated that the catchment area is strongly correlated to both the TN and TP loads (Figure 2). This relationship is even stronger when only catchments having a dominant land cover type are considered (data not shown). Obviously, catchment size is not the only parameter that controls the nutrient loads reaching water bodies, as illustrated by the scattering of data along the relationships between catchment area and TN and TP annual loads. In addition to land cover and land management practices within catchments, the major factors determining the potential for generation of nutrients towards lakes are also related to climatic conditions, lake position in the landscape, catchment geology and stream density. Climate and geology do not display any strong variations within the IDF region and were not included in the model as differences between catchments are expected to be marginal. In addition, stream density and the position of lakes in the IDF landscape are accounted for in the model to modulate the non-parametric relationship between nutrients loads and Chl*a* concentrations/levels in the model. A region-specific validation of the use of catchment size as a proxy of nutrient loads would have been desirable. However, the available data did not allow estimating annual nutrient loads for the 48 lakes included in this study.

### Origin and Current State of Lakes and Reservoirs from the IDF Region

The lakes and reservoirs found in the IDF region are characteristic of urban and periurban areas. Most of these water bodies originated as sand and gravel quarries worked between the 1940s and 1980s or resulted from peat extraction in the mid-19^th^ century. The older water bodies are reservoirs built in the 17^th^ and 18^th^ centuries to provide a reliable water supply for Versailles Castle and the city of Paris. The size of water bodies included in this study range from 5 to 120 hectares, and most of them have a mean depth of less than 3 meters (Table S1). As a consequence of their anthropogenic origin, a very unusual characteristic of these water bodies, compared with natural ones, is that nearly half of them (23 out of 48) are not connected to the hydrological network. The two water body sub-categories (drainage and seepage lakes) have thus very distinct mean catchment sizes (Table 1). Consequently, seepage lakes display significantly lower mean annual Chl*a* concentrations compared with drainage lakes (9.6 and 51.5 µg L^−1^, respectively (p<0.001); Table S1). Though 74% of the observations of drainage lakes indicated a Chl*a* concentration greater than 25 µg L^−1^, only a small proportion (3.3%) of observations of seepage lakes indicated values exceeding this threshold value (Table 2). Overall, 66% of the sampled lakes (32 out of 48) are considered to be eutrophic to hypertrophic (Table S1), which is very similar to other area European countries (e.g., England and Wales [75]).

**Table 1:** Lakes and catchment characteristics of seepage and drainage lakes included in this study.

		Lake area	Lake depth	Catchment area	S ratio	LI cover
		(ha)	(m)	(km^2^)		(%)
Drainage lakes	Min	5.0	0.8	0.16	1.2	0.0
(n = 25)	Max	120.0	4.7	1848.4	20349	76.0
	Median	8.8	1.7	29.5	33.8	9.0
	Mean	18.2	2.0	398.7	1341.8	19.5
	*(sd)*	*(23.9)*	*(0.9)*	*(664.5)*	(4905.3)	*(24.0)*
Seepage lakes	Min	5.2	1.2	0.03	0.42	0.0
(n = 23)	Max	111.5	7.2	23.1	79.2	58.0
	Median	12.3	3.1	0.3	1.2	3.0
	Mean	26.5	3.3	1.4	5.6	11.8
	*(sd)*	*(29.5)*	*(1.4)*	*(4.64)*	*(16.0)*	*(17.5)*

**Table 2:** Responses of IDF lakes and reservoirs to the 2030 policy scenario.

	Seepage lakes		Drainage lakes	
	Mean Chl*a* (µg L^−1^)	% of values >25 µg L^−1^	Mean Chl*a* (µg L^−1^)	% of values >25 µg L^−1^
**Current state**	9.6	3.3	51.5	74.0
**2030 scenario**	6.5	2.2	38.2	72.0
	(−12.2%)	(−33%)	(−11.4%)	(−2.8%)

The figures in brackets correspond to the variation from the current state (in %).

### Sensitivity of IDF Lakes and Reservoirs to Catchment-scale Pressure Changes

The impact of catchment-scale pressure changes (S and LI) was assessed using a quantitative model and a binomial model. Whereas the quantitative model provides more detailed insights regarding the response of lakes to pressure changes, the binomial model may be potentially more relevant to managers to define optimal scenarios to attain specific targets.

#### Quantitative model outputs

**Figure 3:**
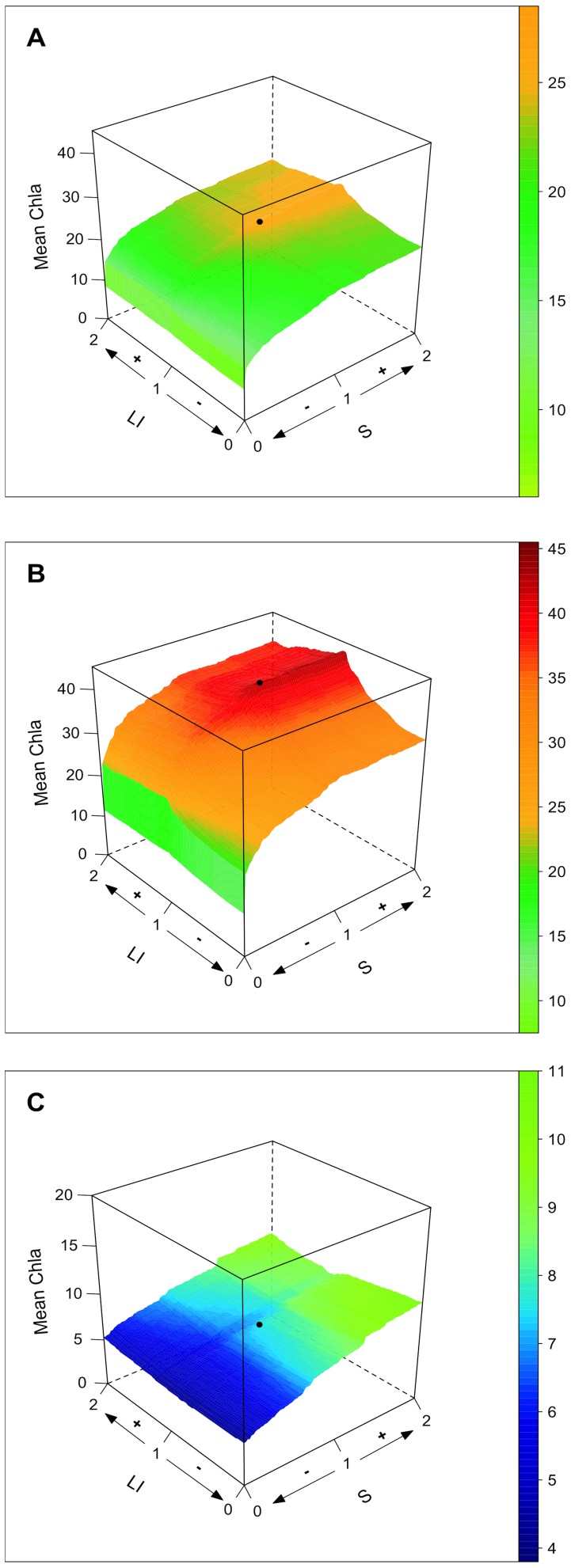
Mean chlorophyll *a* concentration (Mean Chl*a* in µg L^−1^) of water bodies in the IDF region in response to population density (LI; % of urban land cover) and nutrient loads (S; ratio between catchment and water body size). (A) Complete dataset (n = 48), (B) drainage lakes only (n = 25) and (C) seepage lakes only (n = 23). The black dots represent the state at the time of sampling. Population density (LI) and nutrient loads (S) were simulated from zero impact (0) to twice (2) their current state (1).

Using the quantitative model and considering the entire set of water bodies (Figure 3A), we demonstrated that, for the IDF region, the mean Chla concentrations were asymptotically related to changes in nutrient loads (S) and that reducing population density within catchments (LI) is expected to drive an overall reduction of Chl*a* values.

**Figure 4:**
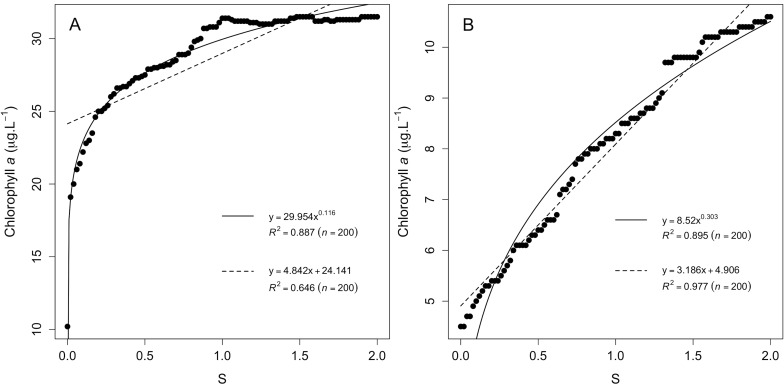
Impact of nutrient loads change (S; ratio between catchment and water body size) on mean chlorophyll *a* concentration. Results obtained for (A) drainage lakes and (B) seepage lakes.

When considering model outputs for drainage (Figure 3B) and seepage (Figure 3C) water bodies separately, the relationship between variations in nutrient loads and mean Chl*a* values for drainage lakes was best represented by a saturating curve (Figure 4A), whereas this relationship was linear for seepage lakes (Figure 4B). These results illustrate the fact that IDF drainage lakes are most likely receiving high nutrient loads compared with seepage lakes and that phytoplankton growth, which is responsible for Chl*a* concentrations, might be limited by other factors than nutrients availability (e.g., light, temperature, residence time) [51]. Thus, whereas drainage lakes will display little improvement until a critical level of pressure reduction is reached, the shape of the response for drainage lakes suggests that the gain that might be obtained may be expected to be proportional to the efforts made on controlling nutrient loads from their catchment. The fact that eutrophic drainage lakes require reaching a critical nutrient load reduction to display a significant decrease in Chl*a* has been reported for other small drainage lakes (e.g., Ravn Lake [28], Loweswater [64], Okaro Lake [76]). However, our results also suggest that seepage lakes may respond in a completely different manner to nutrient load reduction compared with drainage lakes.

**Figure 5:**
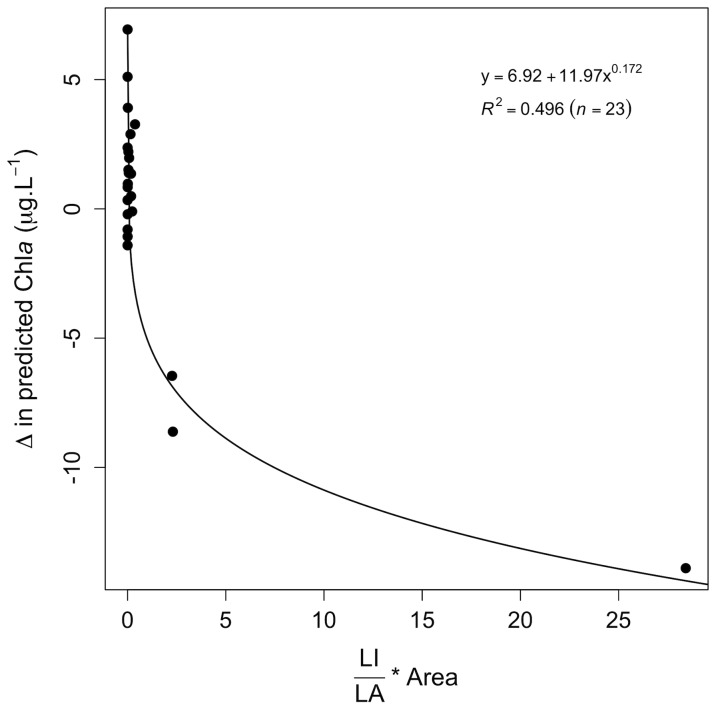
Relationship between the differences in predicted Chl*a* between two contrasting scenarios (“reduced LI” and “increase LI”; see text for details) and the ratio between urban (LI) to agricultural (LA) covers weighted by the catchment size (Area).

In addition, though drainage lakes might benefit from a reduction of population density within catchments (Figure 3B), seepage lakes displayed an unexpected v-shaped relationship between LI and mean Chla concentrations (Figure 3C). To illustrate why a reduction in population density might drive higher mean Chla concentration in some seepage lakes, we analysed the difference in mean Chla (ΔChl*a*) following two contrasting scenarios: (i) a removal of all LI cover within catchments (named hereafter “reduced LI” scenario) and (ii) doubling of LI (named hereafter “increased LI” scenario) (Table 2). Lakes having a ΔChl*a* >0 displayed a greater mean Chl*a* under the “reduced LI” scenario, whereas lakes having a ΔChl*a*<0 displayed a greater mean Chl*a* under the “increased LI” scenario. Figure 5 shows that seepage lakes characterised by small agriculture catchments displayed a higher increase in mean Chl*a* under the “reduced LI” scenario (ΔChl*a* >0), whereas seepage lakes that have larger urbanised catchments would respond more strongly to increasing urban pressure (ΔChl*a*<0). Thus, reducing urban covers in small agricultural seepage lakes catchments may negatively act on their eutrophication status if urban land cover is converted to agricultural land cover. This observation is also consistent with the fact that large catchments are more likely to be exposed to urban point source pollution, whereas smaller catchments are generally mainly subjected to diffuse pollution [75].

#### Binomial model outputs

**Figure 6:**
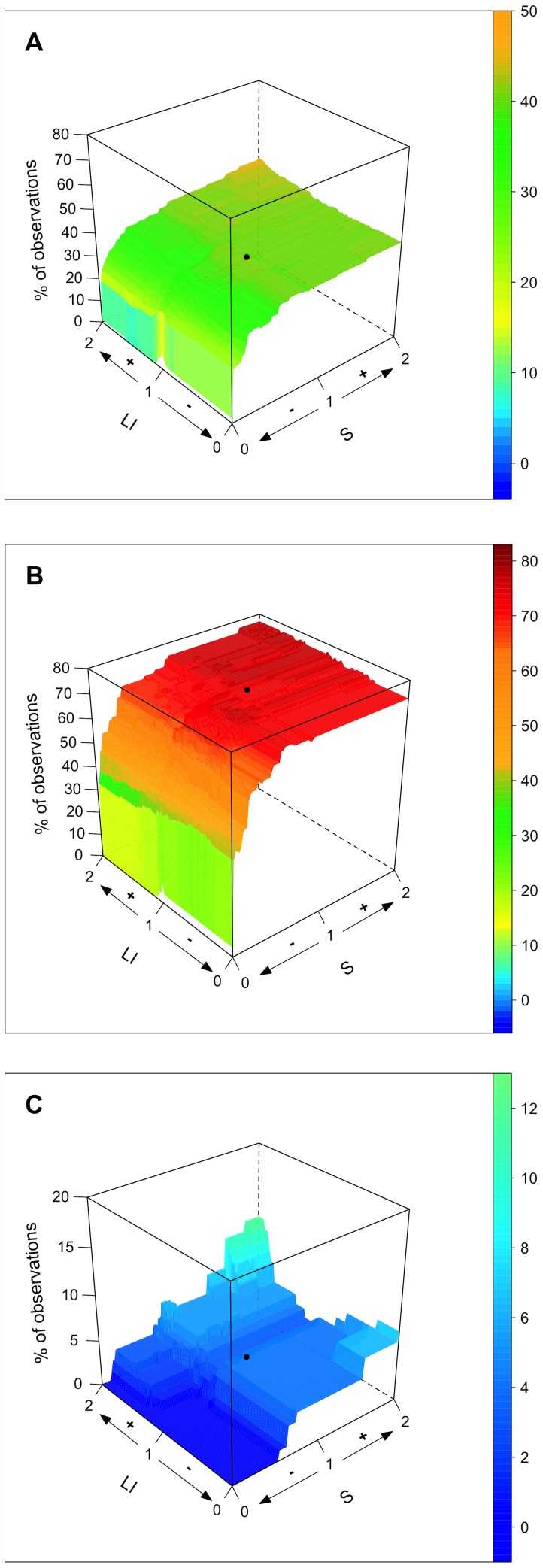
Variation in the frequency of eutrophic water bodies (>25 µg Chl*a* L^−1^ []) in the IDF region in response to population density (LI; % of urban land cover) and nutrient loads (S; ratio between catchment and water body size). (A) Complete dataset (n = 48), (B) drainage lakes only (n = 25) and (C) seepage lakes only (n = 23). The black dots represent the state at the time of sampling. Population density (LI) and nutrient loads (S) were simulated from zero impact (0) to twice (2) their current state (1).

The binomial model outputs (Figure 6) indicated good agreement with the quantitative model outputs. When considering the entire dataset (n = 48 water bodies; Figure 6A), the eutrophication status of water bodies (< or >25 µg L^−1^ Chl*a*) appeared to be relatively insensitive to impervious land cover but asymptotically related to changes in nutrient loads. Despite a doubling of nutrient loads (i.e., a doubling of S), the predicted increase in the frequency of observations over the >25 µg L^−1^ Chl*a* threshold was only approximately 14% (from 77 to 88), whereas a 50% reduction would only be attained if nutrient loads are reduced by 92% (Figure 6A). The asymptotic response to nutrient loads change was even more pronounced for drainage (Figure 6B) because most observations (74%) in this subset of water bodies already displayed Chl*a* values over the >25 µg L^−1^ Chl*a* threshold (Table S1).

Whereas the eutrophication status of drainage lakes and reservoirs appeared to be primarily driven by changes in nutrient loads, isolated (seepage) lakes demonstrated threshold effects in response to both LI and S (Figure 6C). A marked increase in the frequency of eutrophic water bodies (from 4.3 to 12% of observations over the >25 µg L^−1^ Chl*a* threshold) was only obtained after doubling both the LI and S values (worst tested scenario). This is consistent with the quantitative model outputs, where increasing LI was shown to strongly impact the studied seepage lakes characterised by large urbanised catchments.

Again, seepage lakes also appeared to be more prone to positive feedback to reduced pressures. The model predicted that if nutrient loads are reduced by 48% with no change in land cover, all isolated eutrophic water bodies are expected to recover towards a non-eutrophic state. However, an increase in LI would compensate for a reduction in S (for an increase of 68% in LI; see Figure 6C).

### Projecting the 2030 Policy Scenario on IDF Lakes Eutrophication Status

Obtaining a clear picture of how anthropogenic pressures that act on aquatic resources will evolve in the near future is a great challenge and necessarily relies on current expert knowledge. By 2030, nutrient inputs from both point and diffuse sources will likely be reduced by approximately 30% following significant investments aimed at mitigating the impact of past surface water management policies [60]. However, in the meantime, the human population within the Paris area is expected to increase by 20% [10], which was not considered in the nutrient abatement target definition process.

Considering these figures, our models indicate that (i) phytoplankton biomass values of drainage lakes will be reduced by approx. 10% (Chla from 43.1 to 38.2 µg L^−1^), which would have little effect on the frequency of lakes considered as eutrophic, and (ii) the mean Chl*a* of seepage lakes would also be reduced by approx. 10% (from 7.4 to 6.5 µg L^−1^), but the frequency of eutrophic isolated water bodies would be reduced by 30–40% (Fig. 3,6 & Table 2). These figures might not be homogeneously attained over the entire Paris area. However, local projections are not available, and the model outputs only aimed at giving a global estimation of the eutrophication status of periurban lakes by 2030.

The pessimistic forecast provided by our approach illustrated the fact that remediation measures, as currently planned by governmental agencies, are likely to only have a marginal impact on improving the eutrophication status of surface waters, which is unfortunately consistent with scenario analysis in other European countries [75], [77]. If the eutrophication status of surface waters is to be significantly improved, setting costly large-scale (e.g., regional) nutrient abatements targets might be inefficient and should be, at least, complemented or replaced by catchment-specific analyses of nutrient sources associated to appropriate remediation measures [64], [78].

### Model Uncertainty

The uncertainty in models used to forecast the impact of environmental change is a major issue if models are to be used as management tools [64], [68], [79]. The main criticism that may arise from our model lies in the fact that we did not include a direct measure of nutrient loads in the model. However, using literature data from very diverse geographic locations, we could demonstrate that catchment size is a strong predictor of both TN and TP loads (Figure 2). In addition, a significant portion of the unexplained variance in this relationship is most likely due to strong differences in climate, geology and land management practices that are far greater than what is expected in the IDF region.

Our model also does not take into account the potential ‘memory’ effect of lakes due to internal loading of nutrients [80]–[82]. However, most lakes included in the model are relatively young (less than 40 years), small (<120 ha) and shallow (mean depth<5 m). This type of aquatic system has been shown to be highly responsive to external loading reduction [83]–[84], which reinforces the conclusions from our scenarios.

Then, we also only considered the combined effects of two external pressures. In the future, other environmental changes may act as eutrophication drivers. Indeed, climate change will likely have a strong impact on hydrology and diffuse pollution risks [85]. Regarding water quality and availability, the two drivers (nutrients loads and population density) included in this study are expected to change much faster than slow- acting climate change and are known to have turned pristine newly formed artificial lakes into highly eutrophic and degraded habitats within a few decades [86]–[87]. However, further research is still critical to assessing the combined effects of these two fundamental drivers of ecosystem functioning change.

Finally, concerning the 2030 policy scenario selected for the IDF region, it is important to note that it is based on current knowledge, and significant departure from this scenario may occur due to reasons such as unexpected socio-economic factors. However, this scenario is based on conservative assumptions that (i) ongoing efforts in terms of investment in water management will be maintained and (ii) slow economic growth will occur [60].

## Conclusions

Small shallow lakes and reservoirs, which represent the most frequent type of standing water bodies in the world [88], are commonly found in urban and periurban areas. Due to their small size, which results in low inertia regarding both global and local environmental changes [89], these water bodies are particularly vulnerable to eutrophication and catastrophic shifts from a “clear water” state to a turbid state, which is often associated with the proliferation of toxin-producing cyanobacteria [90]. Considering the limited economic resources available to manage these ecosystems, management tools based on models requiring minimal parameterisation may represent a valuable alternative over more complex and data-demanding models. In this paper, we propose a new approach that allows evaluation of how LULC and population density changes might affect the eutrophication status of lakes and reservoirs. This flexible approach can easily be adapted to integrate region-specific environmental contexts but will require further development to be adapted to other types of lentic ecosystems (large and/or deep lakes).

## Supporting Information

Figure S1 Total number of peer-reviewed publication using “climate change” (black bars) or “land-use” change grey bars) in the article title and “freshwater” in the topic.
(DOCX)

Table S1 Model variables and chlorophyll *a* measurements for the 48 lakes included in this study.
(DOCX)

## References

[1] Maltby E, Ormerod S (2011) Freshwaters – Openwaters, wetlands and floodplains. In: UK National Ecosystem Assessment. The UK National Ecosystem Assessment Technical Report. Cambridge: UNEP-WCMC. 295–360. Available: http://uknea.unep-wcmc.org/Resources/tabid/82/Default.aspx. Accessed 31 July 2013.

[2] Maberly SC, Elliott JA (2012) Insights from long-term studies in the Windermere catchment: External stressors, internal interactions and the structure and function of lake ecosystems. Fresh Biol 57: 233–243.

[3] Folt CL, Chen CY, Moore MV, Burnaford J (1999) Synergism and antagonism among multiple stressors. Limnol Oceanogr 44: 864–877.

[4] Ormerod SJ, Dobson M, Hildrew AG, Townsend CR (2010) Multiple stressors in freshwater ecosystems. Fresh Biol (Suppl. 1): 1–4.

[5] Heathwaite AL (2010) Multiple stressors on water availability at global to catchment scales: understanding human impact on nutrient cycles to protect water quality and water availability in the long term. Fresh Biol (Suppl. 1): 241–257.

[6] Elliott JA, May L (2008) The sensitivity of phytoplankton in Loch Leven (UK) to changes in nutrient load and water temperature. Fresh Biol 53: 32–41.

[7] Brookes JD, Carey CC (2011) Resilience to blooms. Science 334: 46–67. 21980099 10.1126/science.1207349

[8] Kareiva P, Watts S, McDonald R, Boucher T (2007) Domesticated nature: Shaping landscapes and ecosystems for human welfare. Science 316: 1866–1869. 17600209 10.1126/science.1140170

[9] Keatley BE, Bennett EM, MacDonald GK, Taranu ZE, Gregory-Eaves I (2011) Land-use legacies are important determinants of lake eutrophication in the anthropocene. PLoS ONE 6: e15913. 21264341 10.1371/journal.pone.0015913 PMC3018476

[10] UN (2012) World Urbanization Prospects: The 2011 revision. New York: United Nations Publications. Available: http://esa.un.org/unpd/wup/index.htm. Accessed 31 July 2013.

[11] Smith VH (2003) Eutrophication of freshwater and coastal marine ecosystems: a global problem. Environ Sci Poll Res 10: 126–139. 10.1065/espr2002.12.142 12729046

[12] European Parliament (2000) Directive 2000/60/EC Establishing a framework for community action in the field of water policy. Brussels: Official Journal of the European Communities L327/1: 1–72. Available: http://eur-lex.europa.eu/LexUriServ/LexUriServ.do?uri=OJ:L:2000:327:0001:0072:EN:PDF. Accessed 31 July 2013.

[13] Dokulil MT, Teubner K (2011) Eutrophication and climate change: present situation and future scenarios. In: Ansari AA, Singh GS, Lanza GR, Rast W, editors. Eutrophication: causes, consequences and control. Dordrecht: Springer Verlag. 1–16.

[14] European Parliament (2007) Towards Sustainable Water Management in the European Union – First stage in the implementation of the Water Framework Directive 2000/60/EC. Brussels: Communication of the European Communities 128: 1–12. Available: http://ec.europa.eu/environment/water/water-framework/implrep2007/index_en.htm#first. Accessed 31 July 2013.

[15] European Environmental Bureau (2010) 10 years of the Water Framework Directive: A Toothless Tiger? Brussels: European Environmental Bureau. 19 p. Available: http://www.eeb.org/index.cfm/library/index.cfm?month=7&year=2010. Accessed 31 July 2013.

[16] Dodds WK, Bouska WW, Eitzmann JL, Pilger TJ, Pitts KL, et al (2009) Eutrophication of U.S. freshwaters: an analysis of potential economic damages. Environ Sci Technol 43: 12–19. 19209578 10.1021/es801217q

[17] Smith VH, Schindler DW (2009) Eutrophication science: Where do we go from here? Trends Ecol Evol 24: 201–207. 19246117 10.1016/j.tree.2008.11.009

[18] UNEP (1994) The pollution of lakes and reservoirs. Nairobi: UNEP Environment Library 10. 36 p.

[19] EEA (2012) CSI 020– Nutrients in freshwater. Available: http://www.eea.europa.eu/data-and-maps/indicators/nutrients-in-freshwater/nutrients-in-freshwater-assessment-published-3. Accessed 31 July 2013.

[20] Elliott JA (2010) The seasonal sensitivity of Cyanobacteria and other phytoplankton to changes in flushing rate and water temperature. Global Change Biol 16: 864–876.

[21] Buisson L, Thuiller W, Lek S, Lim P, Grenouillet G (2008) Climate change hastens the turnover of stream fish assemblages. Global Change Biol 14: 2232–2248.

[22] Olden JD, Jensen OP, Zanden MJV (2006) Implications of long-term dynamics of fish and zooplankton communities for among-lake comparisons. Can J Fish Aquat Sci 63: 1812–1821.

[23] Vollenweider RA (1976) Advances in defining critical loading levels for phosphorus in lake eutrophication. Mem Ist Ital Idrobiol 33: 53–83.

[24] OECD (1982) Eutrophication of waters: monitoring, assessment and control. Paris: OECD Cooperative program on monitoring of inland waters. 154 p.

[25] Jeppesen E, Søndergaard M, Jensen JP, Havens KE, Anneville O, et al (2005) Lake responses to reduced nutrient loading – An analysis of contemporary long-term data from 35 case studies. Fresh Biol 50: 1747–1771.

[26] Carpenter SR, Ludwig D, Brock WA (1999) Management of eutrophication for lakes subject to potentially irreversible change. Ecol Appl 9: 751–771.

[27] Håkanson L, Bryhn AC (2008) A dynamic mass-balance model for phosphorus in lakes with a focus on criteria for applicability and boundary conditions. Water Air Soil Poll 187: 119–147.

[28] Trolle D, Jørgensen TB, Jeppesen E (2008) Predicting the effects of reduced external nitrogen loading on the nitrogen dynamics and ecological state of deep Lake Ravn, Denmark, using the DYRESM–CAEDYM model. Limnologica 38: 220–232.

[29] Beaulac MN, Reckhow KH (1982) An examination of land use – Nutrient export coefficients. Water Resour Bull 18: 1013–1024.

[30] Clesceri NL, Curran SJ, Sedlak RI (1986) Nutrient loads to Wisconsin lakes: Part I. Nitrogen and phosphorus export coefficients. Water Resour Bull 22: 983–990.

[31] Jones JR, Knowlton MF, Obrecht DV, Cook EA (2004) Importance of landscape variables and morphology on nutrients in Missouri reservoirs. Can J Fish Aquat Sci 61: 1503–1512.

[32] Catherine A, Mouillot D, Escoffier N, Bernard C, Troussellier M (2010) Cost effective prediction of the eutrophication status of lakes and reservoirs. Fresh Biol 55: 2425–2435.

[33] Begrström AK, Jansson M (2006) Atmospheric nitrogen deposition has caused nitrogen enrichment and eutrophication of lakes in the northern hemisphere. Global Change Biol 12: 635–643.

[34] Knoll LB, Vanni MJ, Renwick WH (2003) Phytoplankton primary production and photosynthetic parameters in reservoirs along a gradient of watershed land use. Limnol Oceanogr 48: 608–617.

[35] Tetzlaff B, Vereecken H, Kunkel R, Wendland F (2009) Modelling phosphorus inputs from agricultural sources and urban areas in river basins. Environ Geol 57: 183–193.

[36] Fraterrigo JF, Downing JA (2008) Influence of land use on lake nutrients varies with watershed transport capacity. Ecosystems 11: 1021–1034.

[37] Wetzel RG (2001) Limnology – Lake and river ecosystems. Amsterdam: Elsevier Academic Press. 1006 p.

[38] Edmonson WT (1961) Changes in Lake Washington following an increase in the nutrient income. Verh Int Ver Limnol 14: 167–175.

[39] Beaulac MN, Reckhow RH (1982) An examination of land use – nutrient export relationships. Water Resour Bull 18: 1013–1024.

[40] Abell JM, Özkundakci D, Hamilton DP, Miller SD (2011) Relationships between land use and nitrogen and phosphorus in New Zealand lakes. Mar Fresh Res 62: 162–175.

[41] Viessman W, Hammer MJ (1993) Water supply and pollution control. New York: Harper Collins College Publishers. 860 p.

[42] Russell MA, Walling DE, Hodgkinson RA (2001) Suspended sediment sources in two small lowland agricultural catchments in the UK. J Hydrol 252: 1–24.

[43] Walling DE, Fang D (2003) Recent trends in the suspended sediment loads of the world’s rivers. Global Planet Change 39: 111–126.

[44] Frink CR (1991) Estimating nutrient exports to estuaries. J Environ Qual 20: 717–724.

[45] Phillips G, Pietiläinen OP, Carvalho L, Solimni A, Solheim AL, et al (2008) Chlorophyll–nutrient relationships of different lake types. Aquat Ecol 42: 213–226.

[46] Breiman L (2001) Random forests. Mach Learn 45: 5–32.

[47] Cutler RD, Edwards TC, Beard KH, Cutler A, Hess KT, et al (2007) Random forests for classification in ecology. Ecology 88: 2783–2792. 18051647 10.1890/07-0539.1

[48] Young WM, Marston FM, Davis JR (1996) Nutrient exports and land use in Australian catchments. J Environ Manag 47: 165–183.

[49] Bremigan MT, Soranno PA, Gonzalez MJ, Bunnell DB, Arend KK, et al (2008) Hydrogeomorphic features mediate the effects of land use/cover on reservoir productivity and food webs. Limnol Oceanogr 53: 1420–1433.

[50] Huisman J, van Oostveen P, Weissing FJ (1999) Species dynamics in phytoplankton blooms: incomplete mixing and competition for light. Am Nat 154: 46–68. 10.1086/303220 29587494

[51] Reynolds CS (2006) Ecology of phytoplankton. Cambridge: Cambridge University Press. 535 p.

[52] Wasson JG, Chandesris A, Pella H, Blanc L (2002) Typology and reference conditions for surface water bodies in France: the hydro-ecoregion approach. TemaNord 566: 37–41.

[53] Panagos P, Jones A, Bosco C, Senthil Kumar PS (2011) European digital archive on soil maps (EuDASM): preserving important soil data for public free access. Int J Digital Earth 4: 434–443.

[54] Catherine A, Troussellier M, Bernard C (2008) Design and application of a stratified sampling strategy to study the regional distribution of Cyanobacteria (Ile-de-France. France). Water Res 42: 4989–5001. 18945472 10.1016/j.watres.2008.09.028

[55] Scheffer M (2005) Ecology of shallow lakes. Dordrecht: Kluwer Academic Publishers. 374 p.

[56] Catherine A, Escoffier N, Belhocine A, Nasri AB, Hamlaoui S, et al (2012) On the use of the FluoroProbe®, a phytoplankton quantification method based on fluorescence excitation spectra for large-scale surveys of lakes and reservoirs. Water Res 46: 1771–1784. 22280952 10.1016/j.watres.2011.12.056

[57] Guisan A, Zimmermann NE (2000) Predictive habitat distribution models in ecology. Ecol Model 135: 147–186.

[58] Cohen JA (1960) A coefficient of agreement for nominal scales. Educ Psychol Meas 20: 37–46.

[59] Billen G, Garnier J, Hanset P (1994) Modeling phytoplankton development in whole drainage network: the RIVERSTRAHLER Model. Hydrobiologia 289: 119–137.

[60] AESN (2004) Etat des lieux: Bassin Seine et cours d’eau côtiers normands. Paris: Agence de l’Eau Seine Normandie. Available: http://www.eau-seine-normandie.fr/index.php?id=2258. Accessed 31 July 2013.

[61] SDRIF (2008) Schéma directeur de la région Ile-de-France. Paris: Conseil Régional d’Ile-de-France. Available: http://www.iledefrance.fr/missions-et-competences/deplacements-amenagement/le-sdrif/. Accessed 31 July 2013.

[62] Némery J, Garnier J (2007) Origin and fate of phosphorus in the Seine watershed (France): agricultural and hydrographic P budgets. J Geophys Res 11: G03012.

[63] R Development Core Team (2013) R: A language and environment for statistical computing. Vienna: R Foundation for statistical computing. Available: http://www.R-project.org/. Accessed 31 July 2013.

[64] Norton L, Elliott JA, Maberly SC, May L (2012) Using models to bridge the gap between land use and algal blooms: An example from the Loweswater catchment, UK. Environ Model Software 36: 64–75.

[65] Ferguson CA, Carvalho L, Scott EM, Bowman AW, Kirika A (2008) Assessing ecological responses to environmental change using statistical models. J Appl Ecol 45: 193–203.

[66] Wellen C, Arhonditsis GB, Labencki T, Boyd D (2013) Application of the SPARROW model in watersheds with limited information: A Bayesian assessment of the model uncertainty and the value of additional monitoring. Hydrol Process DOI: 10.1002/hyp.9614.

[67] Aspinall R, Pearson D (2000) Integrated geographical assessment of environmental condition in water catchments: Linking landscape ecology, environmental modeling and GIS. J Environ Manage 59: 299–319.

[68] Jakeman AJ, Letcher RA (2003) Integrated assessment and modelling: Features, principles and examples for catchment management. Environ Model Software 18: 491–501.

[69] de Lange WJ, Wise RM, Forsyth GG, Nahman A (2010) Integrating socio-economic and biophysical data to support water allocations within river basins: An example from the Inkomati Water Management Area in South Africa. Environ Model Software 25: 43–50.

[70] Jordan TE, Correll DL, Weller DE (1997) Nonpoint source discharges of nutrients from piedmont watersheds of Chesapeake Bay. J Am W Resources 33: 631–645.

[71] Russell MA, Walling DE, Webb BW, Bearne R (1998) The composition of nutrient fluxes from contrasting UK river basins. Hydrol Proc 12: 1461–1482.

[72] NAWQA (2002) Nutrients National Synthesis Project. Available: http://pubs.usgs.gov/ds/2005/152/htdocs/data_report_data.htm. Accessed 31 July 2013.

[73] Pieterse NM Bleuten W, Jørgensen SE (2003) Contribution of point sources and diffuse sources to nitrogen and phosphorus loads in lowland river tributaries. J Hydrol 271: 213–225.

[74] Kortelainen P, Mattsson T, Finér L, Ahtiainen M, Saukkonen S, et al (2006) Controls on the export of C, N, P and Fe from undisturbed boreal catchments. Aquat Sci 68: 453–468.

[75] Duethmann D, Anthony S, Carvalho L, Spears B (2009) A model-based assessment of non-compliance of phosphorus standards for lakes in England and Wales. JRBM 7: 197–207.

[76] Özkundakci D, Hamilton DP, Scholes P (2010) Effect of intensive catchment and in-lake restoration procedures on phosphorus concentrations in a eutrophic lake. Ecol Engineer 36: 396–405.

[77] Johnes PJ, Foy R, Butterfield D, Haygarth PM (2007) Land use scenarios for England and Wales: evaluation of management options to support ‘good ecological status’ in surface freshwaters. Soil Use Manag 23: 176–194.

[78] Giguet-Covex C, Arnaud F, Poulenard J, Enters D, Reyss JL, et al (2010) Sedimentological and geochemical records of past trophic state and hypolimnetic anoxia in large, hard-water Lake Bourget, French Alps. J Paleolimnol 43: 171–190.

[79] Refsgaard JC, van der Sluijs JP, Højberg AL, Vanrolleghem PA (2007) Uncertainty in the environmental modelling process: A framework and guidance. Environ Model Software 22: 1543–1556.

[80] Sas H (1989) Lake restoration by reduction of nutrient loading. Sankt-Augustin: Academia-Verlag Richarz. p 497.

[81] Jeppesen E, Kristensen P, Jensen JP, Søndergaard M, Mortensen E, et al (1991) Recovery resilience following a reduction in external phosphorus loading of shallow, eutrophic Danish lakes: Duration, regulating factors and methods for overcoming resilience. Mem Ist Ital Idrobiol 48: 127–148.

[82] Phillips G, Jackson R, Bennett C, Chilvers A (1994) The importance of sediment phosphorus release in the restoration of very shallow lakes (The Norfolk Broads, England) and implications for biomanipulation. Hydrobiologia 275/276: 445–456.

[83] Jeppesen E, Jensen JP, Søndergaard M, Lauridsen TL (2005) Response of fish and plankton to nutrient loading reduction in eight shallow Danish lakes with special emphasis on seasonal dynamics. Fresh Biol 50: 1616–1627.

[84] Catherine A, Quiblier C, Yéprémian C, Got P, Groleau A, et al (2008) Collapse of a *Planktothrix agardhii* perennial bloom and microcystin dynamics in response to reduced phosphate concentrations in a temperate lake. FEMS Microbiol Ecol 65: 61–73. 18462398 10.1111/j.1574-6941.2008.00494.x

[85] Heathwaite AL (2010) Multiple stressors on water availability at global to catchment scales: understanding human impact on nutrient cycles to protect water quality and water availability in the long term. Fresh Biol 55: 241–257.

[86] Jinglu W, Chengmin H, Haiao Z, Schleser GH, Battarbee R (2007) Sedimentary evidence for recent eutrophication in the northern basin of Lake Taihu, China: Human impacts on a large shallow lake. J Paleolimnol 38: 13–23.

[87] Schindler DW, Wolfe AP, Vinebrooke R, Crowe A, Blais JM, et al (2008) The cultural eutrophication of Lac la Biche, Alberta Canada: A paleolimnological study. Can J Fish Aquat Sci 65: 2211–2223.

[88] Downing JA, Prairie YT, Cole JJ, Duarte CM, Tranvik LJ, et al (2006) The global abundance and size distribution of lakes, ponds and impoundments. Limnol Oceanogr 51: 2388–2397.

[89] Whitehead PG, Wilby RL, Battarbee RW, Kernan M, Wade AJ (2009) A review of the potential impacts of climate change on surface water quality. Hydrol Sci J 54: 101–123.

[90] Scheffer M, Carpenter S, Foley JA, Folk C, Walker B (2001) Catastrophic shifts in ecosystems. Nature 413: 591–596. 11595939 10.1038/35098000

