# Supplementary material for: Projecting the Impact of Regional Land-Use Change and Water Management Policies on Lake Water Quality: An Application to Periurban Lakes and Reservoirs
Source: PLoS One. 2013 Aug 26;8(8):e72227. doi: 10.1371/journal.pone.0072227 (PMC3753357; doi:10.1371/journal.pone.0072227)
Supplement: Table S1 — Model variables and chlorophyll a measurements for the 48 lakes included in this study. (DOCX) [file pone.0072227.s002.docx]

**Projecting the impact of regional land-use change and water management policies on lake water quality: An application to periurban lakes and reservoirs**

Arnaud Catherine^1*^, David Mouillot^2^, Selma Maloufi^1^, Marc Troussellier^2^ and Cécile Bernard^1^.

^1^ Laboratoire Molécules de communication et adaptation des micro-organismes, Muséum national d’Histoire naturelle, Paris, Ile-de-France, France.

^2^ Laboratoire Écologie des systèmes marins côtiers, Université de Montpellier 2, Montpellier, Hérault, France.

Dr Arnaud Catherine: arnocat@mnhn.fr

Pr David Mouillot: [david.mouillot@jcu.edu.au](mailto:david.mouillot@jcu.edu.au)

Selma Maloufi: [maloufi@mnhn.fr](mailto:maloufi@mnhn.fr)

Dr Marc Troussellier: [troussel@univ-montp2.fr](mailto:troussel@univ-montp2.fr)

Pr Cécile Bernard: [cbernard@mnhn.fr](mailto:cbernard@mnhn.fr)

* Corresponding author. Tel.: +33 (0)140 793 207; Fax: +33 (0)140 793 594. E-mail address: arnocat@mnhn.fr (A. Catherine).

**Keywords:** nature and society interactions; water management; environmental change; anthropogenic pressure, land-use, eutrophication, scenarios, Random Forest, modelling.

**Table S1.** Model variables and chlorophyll *a* measurements for the 48 lakes included in this study.

| **Name** | **S** | **D** | **LI** | **LA** | **LF** | **I_d_** | **Alt** | **HL** | **Chlorophyll *a* (µg L^-1^)** | | | | **EL** |
| --- | --- | --- | --- | --- | --- | --- | --- | --- | --- | --- | --- | --- | --- |
|  |  |  |  |  |  |  |  |  | **min** | **max** | **mean** | **CV%** |  |
| 1- Freneuse Cove | 1.15 | 2.5 | 0.00 | 0.28 | 0.72 | 0.00 | 14.2 | 1 | 16.5 | 161.4 | 91.2 | 66.8 | HE |
| 2- Grand Lavacourt Pond | 0.87 | 4.3 | 0.22 | 0.09 | 0.65 | 0.00 | 14.5 | 0 | 2.4 | 15.1 | 7.7 | 71.9 | M |
| 3- Ilon Cove | 21.8 | 3.1 | 0.04 | 0.08 | 0.43 | 0.00 | 16.2 | 1 | 7.9 | 49.0 | 24.3 | 74.7 | E |
| 4- Les Galets Pond | 0.73 | 2.5 | 0.02 | 0.00 | 0.98 | 0.00 | 24.9 | 0 | 4.8 | 10.3 | 8.0 | 29.9 | ME |
| 5- ASM Club Cove | 5.8 | 2.4 | 0.00 | 0.01 | 0.98 | 0.00 | 19.6 | 1 | 40.3 | 101.3 | 75.4 | 33.9 | HE |
| 6- Grosse Pierre Pond | 0.73 | 4.3 | 0.12 | 0.01 | 0.80 | 0.00 | 19.4 | 0 | 1.1 | 18.6 | 7.1 | 111.7 | ME |
| 7- Bout du Monde Pond | 1 386 | 1.5 | 0.16 | 0.50 | 0.32 | 0.56 | 19.3 | 1 | 0.6 | 29.8 | 9.3 | 148.8 | E |
| 8- Gaule Achéroise Pond | 1.8 | 6.1 | 0.16 | 0.48 | 0.37 | 0.00 | 19.9 | 0 | 2.0 | 17.8 | 7.3 | 97.2 | ME |
| 9- Meaux Park Pond | 1.11 | 1.2 | 0.00 | 0.28 | 0.72 | 0.00 | 44.9 | 0 | 1.1 | 38.7 | 15.9 | 100.9 | E |
| 10- Précy-sur-Marne Pond | 0.90 | 3.3 | 0.02 | 0.37 | 0.61 | 0.00 | 40.1 | 0 | 2.9 | 4.8 | 3.9 | 20.0 | ME |
| 11- Jabline-Anet Lake | 0.82 | 7.2 | 0.08 | 0.00 | 0.82 | 0.00 | 39.5 | 0 | 1.8 | 4.6 | 3.0 | 40.6 | ME |
| 12- Isles-les Villenoy Pond | 1.32 | 2.9 | 0.00 | 0.79 | 0.21 | 0.00 | 44.0 | 0 | 1.8 | 6.1 | 3.7 | 49.6 | ME |
| 13- Lake Inférieur | 15 426 | 1.1 | 0.05 | 0.71 | 0.24 | 0.61 | 44.6 | 1 | 23.3 | 137.1 | 71.2 | 70.9 | HE |
| 14- Les Pâtis Pond | 1.87 | 2.4 | 0.02 | 0.00 | 0.91 | 0.00 | 39.6 | 0 | 4.3 | 19.9 | 11.7 | 56.4 | E |
| 15- UCPA Centre Pond | 1.12 | 5.3 | 0.04 | 0.00 | 0.91 | 0.00 | 39.8 | 0 | 1.7 | 7.3 | 3.7 | 70.0 | ME |
| 16- Loy Pond | 323 | 1.3 | 0.21 | 0.39 | 0.36 | 0.59 | 50.4 | 1 | 47.5 | 126.2 | 79.5 | 46.1 | HE |
| 17- Saint-Cucufa Pond | 11.4 | 2.4 | 0.10 | 0.00 | 0.86 | 1.44 | 114.6 | 1 | 41.1 | 57.3 | 47.1 | 15.1 | HE |
| 18- UTE Louveciennes | 0.42 | 2.3 | 0.00 | 0.00 | 1.00 | 0.00 | 166.8 | 0 | 0.4 | 1.0 | 0.7 | 44.2 | UO |
| 19- Lake Minimes | 29.9 | 1.1 | 0.09 | 0.00 | 0.63 | 0.64 | 52.4 | 1 | 27.3 | 100.3 | 57.2 | 62.4 | HE |
| 20- Swiss Pond | 0.64 | 1.9 | 0.00 | 0.00 | 1.00 | 0.00 | 119.4 | 0 | 2.9 | 18.6 | 7.5 | 101.0 | ME |
| 21- Saint-Quentin Reservoir | 33.8 | 1.7 | 0.22 | 0.37 | 0.34 | 1.40 | 157.4 | 1 | 19.9 | 52.6 | 34.7 | 42.8 | HE |
| 22- Créteil Lake | 6.33 | 4.7 | 0.56 | 0.00 | 0.20 | 0.00 | 34.4 | 1 | 6.3 | 11.7 | 8.6 | 26.6 | E |
| 23- Maurepas Reservoir | 56.6 | 3.0 | 0.66 | 0.03 | 0.29 | 0.31 | 109.4 | 1 | 41.0 | 132.1 | 92.5 | 46.9 | HE |
| 24- Choisy Pond | 7.06 | 2.8 | 0.47 | 0.00 | 0.32 | 0.71 | 34.7 | 0 | 4.9 | 22.5 | 11.1 | 70.1 | E |
| 25- Plage Bleue Pond | 7.02 | 3.2 | 0.49 | 0.00 | 0.29 | 0.00 | 34.4 | 0 | 2.0 | 15.4 | 5.6 | 117.0 | ME |
| 26- Noés Reservoir | 13.1 | 0.8 | 0.54 | 0.08 | 0.27 | 0.79 | 165.2 | 1 | 4.3 | 38.9 | 22.7 | 62.6 | E |
| 27- Saclay Reservoir | 106 | 1.4 | 0.16 | 0.58 | 0.20 | 1.10 | 122.3 | 1 | 23.3 | 40.5 | 30.9 | 23.0 | HE |
| 28- La Veyssière Pond | 18.3 | 2.5 | 0.76 | 0.01 | 0.20 | 0.00 | 34.5 | 1 | 41.9 | 97.0 | 68.2 | 33.3 | HE |
| 29- Vaux-de-Cernay Pond | 657 | 1.4 | 0.11 | 0.38 | 0.48 | 1.07 | 120.5 | 1 | 26.8 | 85.4 | 58.3 | 41.7 | HE |
| 30- Noues de Seine Pond | 79.2 | 3.4 | 0.58 | 0.04 | 0.26 | 0.00 | 34.4 | 0 | 6.4 | 27.9 | 14.6 | 69.3 | E |
| 31- Courcouronnes Pond | 84.4 | 1.6 | 0.65 | 0.01 | 0.27 | 0.00 | 79.4 | 1 | 15.3 | 167.5 | 91.3 | 72.0 | HE |
| 32- Epinoche Pond | 9 452 | 2.5 | 0.09 | 0.48 | 0.41 | 0.55 | 44.2 | 1 | 60.0 | 71.9 | 66.9 | 7.9 | HE |
| 33- Port-Sud Pond | 3 211 | 1.7 | 0.06 | 0.62 | 0.31 | 0.38 | 54.4 | 1 | 19.6 | 68.2 | 40.5 | 50.4 | HE |
| 34- Fontenay/Vic Pond | 13 163 | 1.1 | 0.04 | 0.79 | 0.16 | 0.20 | 49.3 | 1 | 27.3 | 213.2 | 79.1 | 113.5 | HE |
| 35- Fleuri Pond | 36 627 | 2.1 | 0.04 | 0.79 | 0.16 | 0.20 | 49.3 | 1 | 27.9 | 205.0 | 105.1 | 73.6 | HE |
| 36- Seine Port Pond | 14.8 | 2.7 | 0.00 | 0.00 | 1.00 | 0.00 | 39.2 | 0 | 3.3 | 110.0 | 49.8 | 91.4 | HE |
| 37- Vert-le-Petit Pond | 21 804 | 1.3 | 0.04 | 0.79 | 0.16 | 0.20 | 49.3 | 1 | 7.5 | 48.3 | 33.7 | 53.3 | HE |
| 38- Villefermoy Reservoir | 124 | 2.3 | 0.02 | 0.23 | 0.74 | 0.55 | 100.6 | 1 | 27.8 | 41.5 | 32.7 | 18.6 | HE |
| 39- Saint-Blaise Pond | 20349 | 1.3 | 0.04 | 0.77 | 0.19 | 0.25 | 49.4 | 1 | 15.8 | 49.0 | 31.0 | 47.1 | HE |
| 40- Bois-le-Roi pond | 3.81 | 2.5 | 0.07 | 0.01 | 0.35 | 0.00 | 44.4 | 0 | 4.5 | 20.3 | 13.6 | 48.6 | E |
| 41- Samoreau Pond | 1.57 | 2.4 | 0.35 | 0.11 | 0.46 | 0.00 | 44.7 | 0 | 1.5 | 2.5 | 2.0 | 20.8 | O |
| 42- Delomez Pond | 0.53 | 3.9 | 0.00 | 0.03 | 0.97 | 0.00 | 55.0 | 0 | 2.0 | 6.2 | 4.1 | 50.9 | ME |
| 43- Leclerc Pond | 0.89 | 2.6 | 0.00 | 0.00 | 0.70 | 0.00 | 51.6 | 0 | 1.6 | 4.0 | 2.4 | 44.5 | O |
| 44- Grande Paroisse Pond | 1.28 | 3.9 | 0.11 | 0.66 | 0.22 | 0.00 | 50.0 | 0 | 17.8 | 108.7 | 72.0 | 57.2 | HE |
| 45- Grand Marais Pond | 4.23 | 3.3 | 0.07 | 0.60 | 0.33 | 0.00 | 49.8 | 0 | 3.1 | 15.7 | 6.8 | 88.7 | ME |
| 46- Clarette Pond | 1.55 | 1.3 | 0.00 | 0.00 | 1.00 | 0.00 | 54.6 | 0 | 5.5 | 67.4 | 22.5 | 133.8 | E |
| 47- Fromonville Pond | 0.59 | 3.4 | 0.00 | 0.00 | 1.00 | 0.00 | 59.7 | 0 | 6.1 | 8.1 | 7.5 | 12.5 | ME |
| 48- Souppes/Loing Pond | 969 | 3.0 | 0.04 | 0.71 | 0.25 | 0.45 | 66.0 | 1 | 3.9 | 22.3 | 11.0 | 72.9 | E |

**Model parameters:** S: Catchment to waterbody surface area ratio; D: mean (n=3) depth of waterbody (m); LI. LA and LF: Percentage of catchment land use classified as impervious urban, agriculture, and forest respectively (%); Id: drainage index (km^-1^); Alt: altitude (m) of waterbodies; HL: lakes connected (1) and or not connected (0) to streams.

EL: eutrophication level according to the fix boundaries of OECD (1982) with UO: ultra-ologotrophix; O: oligotrophic; ME: mesotrophic; E: eutrophic and HE: hypertrophic.
