# Supplementary material for: Projecting the Impact of Regional Land-Use Change and Water Management Policies on Lake Water Quality: An Application to Periurban Lakes and Reservoirs
Source: PLoS One. 2013 Aug 26;8(8):e72227. doi: 10.1371/journal.pone.0072227 (PMC3753357; doi:10.1371/journal.pone.0072227)
Supplement: Figure S1 — Total number of peer-reviewed publication using “climate change” (black bars) or “land-use” change grey bars) in the article title and “freshwater” in the topic. (DOCX) [file pone.0072227.s001.docx]

**Projecting the impact of regional land-use change and water management policies on lake water quality: An application to periurban lakes and reservoirs**

Arnaud Catherine^1*^, David Mouillot^2^, Selma Maloufi^1^, Marc Troussellier^2^ and Cécile Bernard^1^.

^1^ Laboratoire Molécules de communication et adaptation des micro-organismes, Muséum national d’Histoire naturelle, Paris, Ile-de-France, France.

^2^ Laboratoire Écologie des systèmes marins côtiers, Université de Montpellier 2, Montpellier, Hérault, France.

Dr Arnaud Catherine: arnocat@mnhn.fr

Pr David Mouillot: [david.mouillot@jcu.edu.au](mailto:david.mouillot@jcu.edu.au)

Selma Maloufi: [maloufi@mnhn.fr](mailto:maloufi@mnhn.fr)

Dr Marc Troussellier: [troussel@univ-montp2.fr](mailto:troussel@univ-montp2.fr)

Pr Cécile Bernard: [cbernard@mnhn.fr](mailto:cbernard@mnhn.fr)

* Corresponding author. Tel.: +33 (0)140 793 207; Fax: +33 (0)140 793 594. E-mail address: arnocat@mnhn.fr (A. Catherine).

**Keywords:** nature and society interactions; water management; environmental change; anthropogenic pressure, land-use, eutrophication, scenarios, Random Forest, modelling.

**Figure S1.** Total number of peer-reviewed publication using “climate change” (black bars) or “land-use” change grey bars) in the article title and “freshwater” in the topic. Data from Web of Knowledge (10^th^ February 2013).
